# Phylogeographic History of *Atraphaxis* Plants in Arid Northern China and the Origin of *A*. *bracteata* in the Loess Plateau

**DOI:** 10.1371/journal.pone.0163243

**Published:** 2016-09-22

**Authors:** Zhe Xu, Ming-Li Zhang, James I. Cohen

**Affiliations:** 1 Key Laboratory of Biogeography and Bioresource in Arid Land, Xinjiang Institute of Ecology and Geography, Chinese Academy of Sciences, Urumqi, 830011, China; 2 State Key Laboratory of Systematic and Evolutionary Botany, Institute of Botany, Chinese Academy of Sciences, Beijing, 100093, China; 3 Applied Biology, Kettering University, 1700 University Ave., Flint, MI, 48504, United States of America; Technical University in Zvolen, SLOVAKIA

## Abstract

In China, species of *Atraphaxis* (Polygonaceae) primarily inhabit arid zones across temperate steppe and desert regions. The complex geologic history (e.g., expansion of deserts) and extreme climate shifts of the region appear to have played an important role in shaping the phylogeography of *Atraphaxis*. The present study focuses on species-level phylogeographic patterns of *Atraphaxis* in China, with the goal of determining the impact of past environmental changes, in northern China, on the evolutionary history of the genus. Five hundred and sixty-four individuals distributed among 71 populations of 11 species of *Atraphaxis* from across the geographic range of the genus were studied using sequence data from two plastid spacers, *psb*K-*psb*I and *psb*B-*psb*H. The results demonstrate that most chloroplast haplotypes are species-specific, except for some present among widespread species. The phylogeny of *Atraphaxis* was well structured, and molecular dating analyses suggest that the main divergence events occurred during the late Pliocene and Pleistocene (5.73–0.03 million years ago). The statistical dispersal-vicariance analysis (S-DIVA) results provide evidence that phylogeographic patterns for the genus were characterized by both vicariance events and regional dispersal. The presented data suggest that the rapid expansion of deserts and climatic changes in northern China during the late Pliocene and Pleistocene have driven the diversification and spread of *Atraphaxis* in the region. The expansion of the Tengger Desert provided appropriate conditions for the origin of *A*. *bracteata*. Additionally, a contact zone in the north of the Hexi Corridor was identified as having played a significant role as a migratory route for species in adjacent areas.

## Introduction

The Pleistocene was characterized by extensive oscillations of the global climate that strongly affected the evolution and geographic distributions of organisms and communities [1,2]. The effects of these Pleistocene climate oscillations on phylogeographic patterns throughout temperate alpine and high-latitude regions have been well supported [3]. However, the impact of these oscillations remains poorly explored on the phylogeographic patterns of species in arid areas. The limited number of phylogeographic studies suggest that Pleistocene climatic cycles associated with habitat fragmentation are important environmental factors that shaped the dynamic distribution and genetic patterns of drought-resistant desert plants in the northern hemisphere [4–6]. The few studies that have been conducted on the impact of Pleistocene glaciations in arid regions provide evidence that desert plants suffered from increased aridity and lower temperatures [2,7], and, as a result, suitable habitat for these plants was reduced and fragmented, which promoted allopatric divergence among isolated populations and, in some instances, drove speciation [7]. Throughout periods of glaciation, glacial refugia have served as important reservoirs for biological diversity [8,9], and during interglacial periods when the climate became warmer and more humid, many lineages left glacial refugia and recolonized and diversified into newly available habitats [10]. Thus, the climatic oscillations during the Pleistocene helped drive species diversification and migration, processes that can be critically examined using phylogeographic analyses to better understand the patterns of spatial arrangements and genetic history of species [11,12]. Phylogeographic studies describe historical changes at spatial and temporal scales that impact patterns of gene flow, isolation, and migration within a species or group of closely related species. These studies can provide evidence for the location of glacial refugia and routes of colonization and range expansion after glacial periods [13–17].

Northern China, part of arid central Asia, has regional variation in aridity, ranging from the typical arid region of northwestern China (annual precipitation ≤200 mm) to the eastern semi-arid sandlands (annual precipitation ≤400 mm) [18]. The arid conditions prevailing over much of the region date back to the early Miocene, and these conditions were strongly influenced by both the uplift of the Qinghai-Tibet Plateau (QTP) and the westward regression of the Palaeo-Mediterranean [19–21]. The uplift of the QTP obstructed sea breezes, from the Indian Ocean, from reaching northern China, and this resulted in the East Asian monsoons from the Pacific Ocean restricting precipitation far inland. Consequently, the decreased precipitation accelerated the formation of ancient deserts, such as the Taklimakan, Gurbantunggut and Badain Jaran-Tengger deserts [22]. During the Pleistocene, these deserts increased in aridity and expanded dramatically [23]. Computer simulations provided additional evidence that the expansion of deserts in China during the Quaternary glacial periods arose via a decrease in the strength of the horizontal winds, enhanced atmospheric subsidence, and reduced moisture transport [22]. Additionally, rapid orogenic movements in the Tianshan and other mountain ranges during the Pleistocene caused local rain shadows and promoted extreme local aridity in some areas [22]. Thus, the extensive perturbations of the climate coupled with geological movements during the Pleistocene sharply increased aridity in northwestern China [24,25].

Despite the dry conditions, arid northern China exhibits outstanding floristic diversity, with many species endemic to the area [26,27]. Plant diversity in the arid core of northern China is largely restricted to the alpine melt ecosystems and areas of sporadic seasonal precipitation. From the perspective of phytogeographical studies, arid northwestern China belongs to the “Irano-Turanian Region” [28]. Except for some ancient relict species (e.g., *Potaninia*, *Tetraena*) [18], evidence suggests most species (e.g., *Salsola*, *Tamarix*, *Zygophyllum*) originated and migrated from central Asia. The eastern sandlands of this region are part of the “Mongolia Flora” and species that inhabit the sandlands are hypothesized to have primarily migrated from Mongolia and East Asia [18].

Both increased aridification and range contraction and isolation during dry/cold episodes in glacial cycles might have played a major role in promoting population divergence and speciation in arid northern China. Genetic evidence of population fragmentation is available for *Tetraena mongolica* Maxim., *Gymnocarpos przewalskii* Maxim., *Lagochilus* Bunge ex Benth., and *Atraphaxis frutescens* (L.) K. Koch [22,29–31], and these arid plants currently have limited distributions in Taklimakan Desert, Hexi Corridor, and Alashan desert. Very little is known concerning the response to past environmental changes of arid species that have continuous geographic distributions across northern China. Given the small amount of available data, it can be postulated that these plant species were affected by climate changes during glacial cycles, and these plants followed migratory routes in response to changes in the climate; however, these postulations have yet to be tested.

To better understand the impact of these environmental changes on the diversification of arid northern China, we examined the evolutionary history of *Atraphaxis* L. (Polygonaceae). The genus includes approximately 25 species primarily distributed throughout northern Africa and western and central Asia [32,33]. In arid northern China, *Atraphaxis* is one of the most representative and diverse plant genera, with ca. 11 species (*A*. *laetevirem*, *A*. *jrtyschensis*, *A*. *pungens*, *A*. *pyrifolia*, *A*.*decipiens*, *A*. *frutescens*, *A*. *bracteata*, *A*. *compacta*, *A*. *canescens*, *A*. *spinosa*, *A*. *manshurica*) [32] distributed in the deserts of northwestern China as well as extending eastwards into the semi-arid monsoon region of eastern China. Previous studies [32,33] have suggested that *Atraphaxis* originated in central Asia, with a few species expanding to northern China. In China, *Atraphaxis* primarily occurs in northern China, including ten species in the arid northwestern part of the country and one species, *A*. *manshurica*, distributed in the semi-arid Horqin sandlands [32]. Three species in the genus present in northern China, *A*. *jrtyschensis*, *A*. *bracteata* and *A*. *manshurica*, are endemic to the country.

*Atraphaxis* is highly drought-tolerant and inhabits areas along the foothills of mountains and edges of deserts [33]. Recent studies of phylogenetic relationships within the genus, using chloroplast DNA (cpDNA) and nuclear ribosomal DNA regions [34,35], suggest that *Atraphaxis* is monophyletic, but the patterns of temporal and geographical diversification remain poorly understood. The present study aims to investigate these patterns, with the goal of providing a more comprehensive historical perspective on both the biota and geological evolution of species in the arid northern China.

To tackle this project, we employed cpDNA sequence data to infer genetic patterns and population responses of species of *Atraphaxis* to past environmental changes throughout arid northern China. The palaeoclimatic scenario proposed for the region by Meng and Zhang [22] will allow for an interpretation of phylogeographic patterns, derived from molecular markers, in a specific environmental context. Together, this information will help determine the impact of past environmental changes, in northern China, on species of *Atraphaxis*.

## Material and Methods

### Sample collection

**Fig 1:**
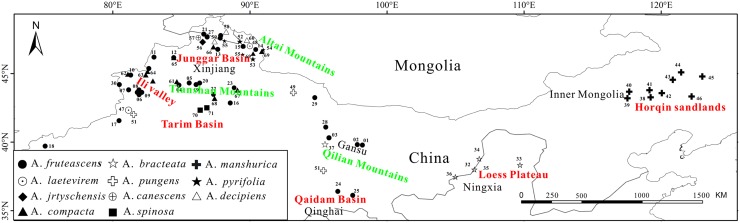
Sampling locations and distributions of populations in 11 species of *Atraphaxis* in arid northern China. Population codes are consistent with population names in Table 1.

A total of 564 individuals representing 11 species of *Atraphaxis* were sampled from 71 populations throughout northern China (Table 1, Fig 1). The sampling included: *A*. *jrtyschensis* from Burqin County in Xinjiang Province; *A*. *bracteata* collected in sandy areas of Inner Mongolia, Ningxia, Shaanxi and Gansu Province; *A*. *manshurica* from the Horqin sandy and its adjacent regions; the dominant species, *A*. *frutescens*, sampled from throughout Xinjiang, Gansu, Qinghai Province; and seven species (*A*. *canescens*, *A*. *compacta*, *A*.*decipiens*, *A*. *laetevirem*, *A*. *pungens*, *A*. *pyrifolia*, and *A*. *spinosa*)_sampled in and around the Tianshan and Altai Mountains. This sampling scheme encompassed most of the natural geographic distribution of each species in China. In previous phylogenetic analyses of Atraphaxideae (Polygonaceae) based on five cpDNA regions, species of *Atraphaxis* in China were resolved as a well-supported monophyletic group [36]. Sample size per population ranged from two to 18 individuals, according to population density. The latitude and longitude of each locality were recorded using a global positioning system (GPS). Silica-dried tissues (leaves and/or flowers from each individual) were collected for DNA extraction. Voucher specimens for all 71 populations are deposited in the Herbarium of the Xinjiang Institute of Ecology and Geography, Chinese Academy of Sciences (XJBI). Our study did not concern Human Subject Research or Animal Research. We can confirm that the leaf materials did not come from conservation parks, and none of the samples involved endangered or protected species

**Table 1:** Details of *Atraphaxis* populations in study, sample sizes and cpDNA haplotypes observed.

Species	n	h(±SD)	π(±SD) (×10^−3^)	mean number of pairwise differences
*A*. *frutescens*	256	0.8365±0.0080	105.211±55.310	6.4178±3.050
*A*. *bracteata*	40	0.3576±0.0911	23.992±16.387	1.5115±0.9292
*A*. *manshurica*	104	0.0752±0.0354	2.567±3.809	0.1720±0.2304
*A*. *laetevirem*	13	0.6154±0.0782	11.349±10.310	0.6923±0.5595
*A*. *pungens*	22	0.6277±0.0602	29.806±19.897	1.8182±1.0878
*A*. *pyrifolia*	25	0.5267±0.0836	80.098±44.464	5.4467±2.7132
*A*. *jrtyschensis*	5	0	0	0
*A*. *canescens*	2	0	0	0
*A*. *decipiens*	16	0.5417±0.0985	54.645±33.148	3.3333±1.8052
*A*. *compacta*	66	0.4103±0.0709	39.213±23.797	2.4704±1.3522
*A*. *spinosa*	15	0	0	0
Total	564	0.8969±0.0069	7.234±3.748	7.3712±3.4531
West region	357	0.9138±0.0055	7.054±3.666	7.1883±3.3775
Central region	103	0.5877±0.0368	127.597±65.858	8.5490±3.9840
East region	104	0.0390±0.0267	2.049±3.368	0.1373±0.2038

### DNA extraction, amplification and sequencing

Total genomic DNA was extracted, using a modified CTAB method [37], from silica-dried tissues of the 564 individuals of *Atraphaxis*. To assess phylogeographic patterns, we amplified and sequenced two cpDNA intergenic spacers, *psb*K-*psb*I [38] and *psb*B-*psb*H [39]. Each PCR amplification was implemented in a 30 μl volume including 2 μl of 10×PCR reaction buffer, 2 μl of 25mM MgCl_2_, 1.8 μl of each primer at 50 ng/μl, 2.0 μl of 2.5mM dNTP, 0.5 μl of Taq DNA-polymerase, and 1μl of genomic DNA at 60 ng/μl. The cycling procedures of the reaction were as follows: initial denaturation at 95°C for 4 minutes (min), followed by 32 cycles of 30 seconds (s) at 94°C, 30 s at 52°C, 60 s at 72°C, and a final extension step of 10 min at 72°C. PCR products were visualized on agarose gels and purified using an ethanol precipitation prior to sequencing [40].

Sequencing was carried out by Sangon Biotech (Shanghai) Co., Ltd., China using an ABI 3730XL automatic sequencer (Applied Biosystems, Foster City, CA, USA). Sample sequences were edited in Seqman (Lasergene, DNASTAR Inc., Madison, Wisconsin, USA), aligned using CLUSTAL_W [41], and then adjusted manually to ensure accurate allelic sequences. All obtained sequences are deposited in GenBank with accession numbers KM452785-KM452792, KX236017-KX236033, KR183867, KR183868, KR183870 for *psb*K-*psb*I; and KM452793-KM452796, KR183863, KR183864, KR183866, KX236034-KX236045 for *psb*B-*psb*H (S2 Table).

### Analysis of genetic diversity

The *psb*K-*psb*I and *psb*B-*psb*H sequences were concatenated for analysis. DnaSP v. 5.0 [42] was utilized to estimate nucleotide polymorphism at all sites in a sequence. For the sequence data set, we calculated haplotype diversities (*h*) and nucleotide diversities (π) using ARLEQUIN 3.5 [43]. These measurements of molecular diversity reflect both the overall number of haplotypes and their molecular divergence [9]. We subdivided the distribution range into three approximately equal geographical regions based on mean humid conditions, and these closely corresponded to longitudinal regions: West (80–95°E, includes the Tarim and Junggar Basin; extremely arid conditions), Central (95–115°E, includes the Hexi Corridor, Alashan mountains, and Muus desert; arid conditions), and East (115–125°E, includes the Horqin sandlands; semi-arid conditions). Genetic variation in different regions and species was estimated using AMOVA, as implemented in ARLEQUIN 3.5, with significance tested via 10,000 permutations [44]. Permut version 1.0 [45] was employed to estimate within-population diversity (*G*_ST_, *N*_ST_) and total gene diversity (*H*_S_, *H*_T_), using 10,000 permutations. The *G*_ST_ and *N*_ST_ coefficients estimate the ratio between the mean within-population genetic diversity and total genetic diversity. While the *G*_ST_ index makes use only of the allelic frequencies, *N*_ST_ also takes into account the genetic distances among haplotypes. A higher *N*_ST_ signifies that more closely related haplotypes occur in the same population, and this indicates the presence of phylogeographic structure [45].

To test for demographic history, the mismatch distribution and neutrality tests (Tajima’s *D* and Fu’s *Fs*) were performed using ARLEQUIN 3.5. The significance of these tests was evaluated as the proportion of random statistics less than or equal to the observed value. For the mismatch distribution, a “ragged” multimodal distribution generally indicates that a population is in demographic equilibrium, whereas a smooth unimodal distribution is associated with recent demographic or range expansion [46]. We tested hypotheses of expansion for all populations of *Atraphaxis* as well as for populations for each species. In addition, in order to take full advantage of historical signals within DNA sequences, the estimates of changes in demographic growth over the history of major areas and the historical demographic dynamics of *Atraphaxis* were inferred via Extended Bayesian skyline plot (EBSP) analyses using BEAST version 1.5.4 [47]. The EBSP analyses are useful because two chloroplast sequences are employed to estimate effective population size through time. Linear and stepwise models were explored using an uncorrelated lognormal relaxed clock. Runs consisted of 10,000,000 generations, with trees sampled every 1,000 generations. The EBSP was visualized in the program Tracer version 1.5.

### Bayesian divergence time estimations and network representation

Approximate divergence times among haplotypes were estimated using a Bayesian log-normal relaxed clock method, as implemented in BEAST version 1.5.4 [47]. We used the Bayesian Information Criterion (BIC) to estimate the best substitution model for the BEAST analyses, which was determined via Modeltest version 3.7 [48]. We used a coalescent model, a GTR substitution model with a Gamma site heterogeneity model, and the uncorrelated log-normal relaxed clock. Due to the lack of fossil data for the genus, along with no specific substitution rates available, we used a range (1.0–10.0×10^−3^ s/s/million) of nucleotide evolution for the genus to estimate divergence times [49,50]. The MCMC simulation was run for 10,000,000 generations, with trees sampled every 1,000 generations and the first 20% of generations were discarded as burnin. Two independent runs achieved the same results. The results were viewed in Tracer version 1.5 to check for convergence to a stationary distribution and for an effective sample size (ESSs) >200. Logs and tree files were combined in Logcombiner version 1.5.4 and summarized in Treeanotator 1.5.4 [47]. Final trees were viewed and edited in FigTree version 1.3.1 (http://tree.bio.ed.as.uk/software/figtree/). The phylogenetic tree was reconstructed without the inclusion of some individuals from species (populations FYD, QKE, SRBP, ALT) that had overlapping distributions as well as widespread species (species *A*. *frutescens* and *A*. *compacta*; see S3 Table). This approach was undertaken to reduce the impact of introgression on the resulting phylogeny [18].

**Fig 2:**
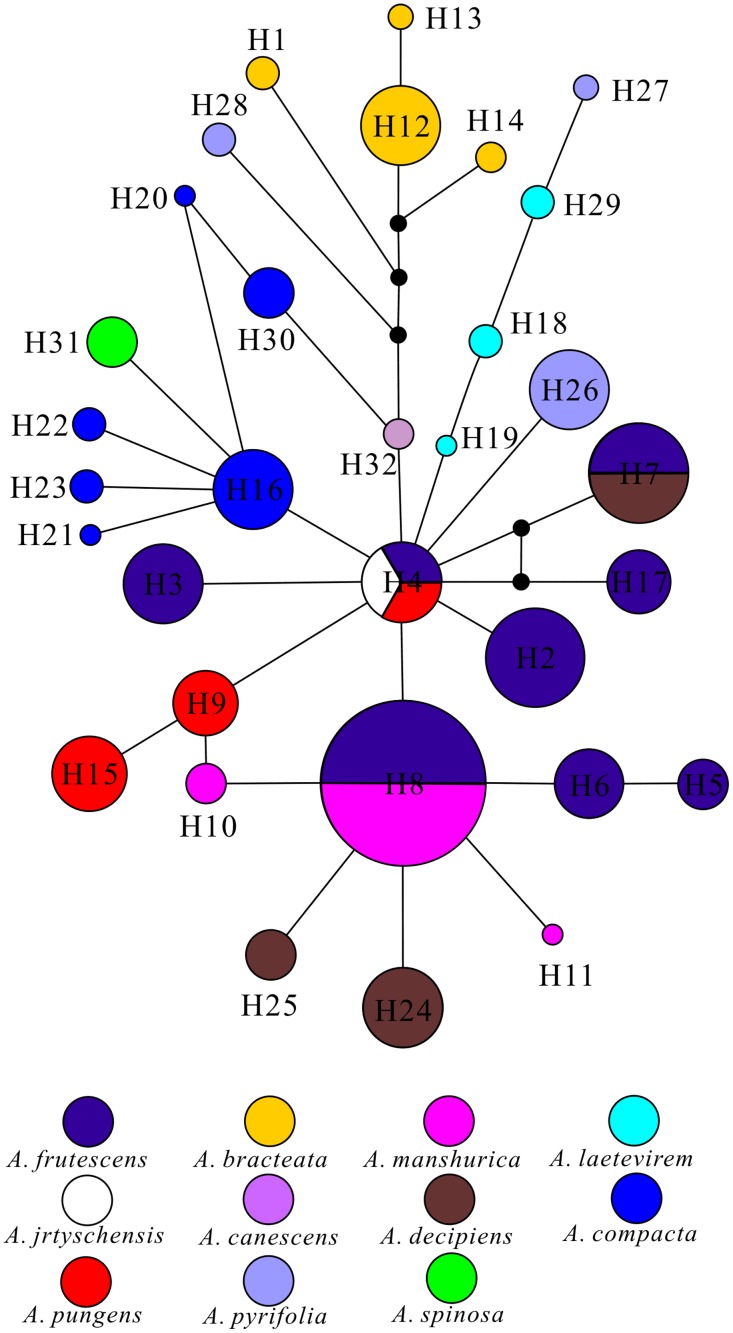
Median-joining network of 32 haplotypes of *Atraphaxis* sampled in northern China. Each circle (H1-H32) represents a unique haplotype, with circle size reflecting haplotype frequencies. Black circles indicate potentially unsampled or extinct haplotypes. The colors within each species are consistent in all Figures.

To investigate relatedness among haplotypes, we resolved a median-joining (MJ) network using Network version 4.6.0.0 [51]. This network can display genealogical relationships and reticulation among haplotypes that tree-building methods may not detect (Fig 2).

### Ancestral area optimization

Although many biogeographic analyses have used dispersal-vicariance analysis (DIVA) [52] to infer ancestral distributions [53,54], DIVA requires fully bifurcated trees [55]. Because our Bayesian trees were not fully resolved, we performed statistical dispersal-vicariance analysis (S-DIVA) to reconstruct the geographic diversification of *Atraphaxis*, as implemented in RASP version 2.0 (http://mnh.scu.edu.cn/soft/blog/RASP), which allows for uncertainties in phylogenetic trees [56]. Six geographic regions were identified for species of *Atraphaxis* in China: (A) Ili valley; (B) Loess Plateau; (C) Horqin sandlands; (D) Junggar Basin; (E) Tarim Basin; (F) and Qaidam Basin (Fig 1). We used 9,000 trees from the Bayesian phylogenetic analyses as input for S-DIVA to compute the condensed tree, and subsequently the ancestral areas and potential vicariance and dispersal events were inferred with RASP.

## Results

### Characteristics of cpDNA regions

The total aligned lengths of the *psb*K-*psb*I and *psb*B-*psb*H intergenic spacers for all species of *Atraphaxis* are 424 bp and 595 bp, respectively, with a total of 70 variable characters, including 31 structural characters (i.e., gaps) coded using simple gap coding [57]. The combined chloroplast intergenic spacers are A/T rich (64.98%), and this nucleotide composition is similar to that of most noncoding spacers and pseudogenes because of low functional constraints. From all of the variable sites, a total of 32 haplotypes (H1-H32) were identified, and the distribution and frequency of each haplotype is presented in Table 1, S1 Table, Fig 1 and S1 Fig.

Thirteen of the 32 haplotypes were identified in more than two populations, with haplotype frequencies ranging from less than 1% to 24% (Table 1). Nineteen haplotypes were very rare and highly localized, as they occurred in only one or two closely associated localities (Table 1, Fig 1 and S1 Fig). These haplotypes were concentrated in the western region (Xinjiang province), with four in the central region and one in the eastern region of the Horqin sandlands. The most widely distributed haplotypes were H8, H4 and H3. Indeed, 16 populations throughout the western and eastern regions of the genus in northern China share haplotype H8. Haplotype H4 was present in seven populations, primarily occurring in the Ili valley and Junggar Basin. Haplotype H3 was distributed among nine populations present in the lower montane zones of the central and east Tianshan range. Some haplotypes were present in only a single species, while other haplotypes were represented among widespread species (e.g., *A*. *frutescens* and *A*.*compacta*). In some instances, several species that are closely related phylogenetically or geographically share the same haplotype (Table 1 and S1 Fig). For example, haplotype H4 was shared by *A*. *frutescens*, *A*. *jrtyschensis*, and *A*. *pungens*; H7 was shared by *A*. *frutescens* and *A*. *decipiens*; and haplotype H8 was shared by *A*. *frutescens* and *A*. *manshurica*.

### Phylogeographic patterns in *Atraphaxis*

The cpDNA sequences showed considerable variation in *Atraphaxis*. For the genus, data on haplotype diversity, as measured by two different indices, gene diversity (*H*) and molecular diversity (*π*), are presented in Table 1. Total genetic diversity (*h*_T_) was 0.919 (±0.0152), and within-population diversity (*h*_S_) was 0.113 (±0.0242).

In the three defined geographic regions, genetic diversity in the western region was much greater than that in the others (Table 1). Total genetic variation was partitioned by species and by geographic region (Table 2). In analyses based on species, the results demonstrated that 47.25% of the total genetic diversity was attributed to differences among species while a similar amount, 46.36%, was due to differences among populations. Only a small quantity, 6.38%, was attributed to differences within populations. In analyses based on geographic regions, variation among populations occupied the largest quantity, 72.11%, with variation among regions accounting for a smaller amount, 21.34%. As with total genetic diversity by species, within population variation was quite small, 6.55% (Table 2). Permut calculated a high *N*_ST_ (0.909), and this was substantially greater than *G*_ST_ (0.877), indicating obvious population differentiation and suggesting the existence of phylogeographic structure for all populations.

**Table 2:** Analysis of molecular variance of *Atraphaxis* populations, partitioned by species and geographic region.

Partitioning	Source of variation	d.f	Sum of squares	Variance components	Percentage of variation
By species	Among species	10	919.467	1.901	47.25
	Among populations	60	908.493	1.865	46.36
	Within populations	488	125.286	0.257	6.38
	Total	558	1953.247	4.022	
By region	Among regions	2	315.719	0.836	21.34
	Among populations	68	1512.242	2.826	72.11
	Within populations	488	125.286	0.257	6.55
	Total	558	1953.247	3.919	

**Fig 3:**
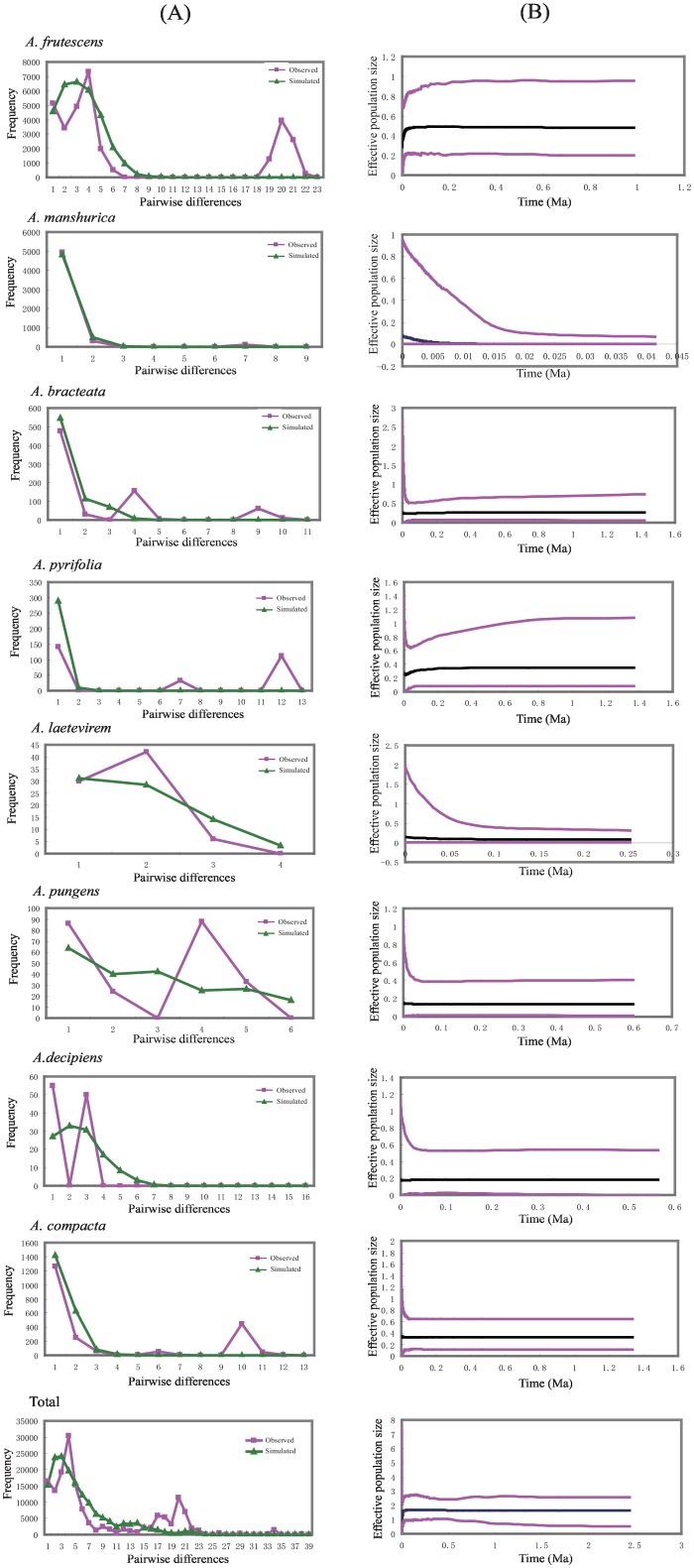
Historical demography for each species and total samples inferred from cpDNA sequences. (A) Mismatch distribution analysis for each species and total samples. (B) Extended Bayesian skyline plot (EBSP) analyses for each species and total samples, showing effective population size as a function of time.

The observed mismatch distribution analysis for the total samples revealed similar results with those of the Extended Bayesian Skyline Plots (EBSP) analyses. The mismatch distribution for all samples was multimodal (Fig 3). For the more conservative neutrality estimates, Tajima’s *D* (*D* = -1.563, P<0.05) was significant and Fu’s *Fs* was not (*Fs* = 0.308, Table 3). The mismatch distribution analysis performed for each species resulted in multimodal distributions. Combined with the non-significant distribution of the EBSP analysis and unapparent neutrality estimates (Fig 3), the results indicated that the populations for each species did not depart from equilibrium.

**Table 3:** Results of Tajima’s *D* and Fu’s *Fs* tests and mismatch analyses for 11 species of *Atraphaxis*.

Tajima’s *D* test			Fu’s *Fs* test		Mismatch distribution		
Species	D	P	Fs	P	τ	θ_0_	θ_1_
*A*. *frutescens*	0.835	0.819	13.119	0.983	3.176	0.000	5.933
*A*. *manshurica*	-0.802	0.205	-1.331	0.159	3.000	0.000	0.073
*A*. *bracteata*	-1.485	0.055	1.852	0.845	3.000	0.000	0.430
*A*. *pyrifolia*	0.168	0.622	9.349	0.999	0.000	0.000	99999.000
*A*. *laetevirem*	0.208	0.662	0.049	0.438	0.895	0.000	99999.000
*A*. *jrtyschensis*	0.000	1.000	-	-	-	-	-
*A*. *pungens*	1.845	0.972	3.043	0.924	4.227	0.000	2.967
*A*. *decipiens*	0.007	0.546	4.747	0.978	2.047	0.000	3.600
*A*. *compacta*	-0.384	0.400	2.412	0.844	3.000	0.000	0.546
*A*. *canescens*	0.000	1.000	-	-	-	-	-
*A*. *spinosa*	0.000	1.000	-	-	-	-	-
Total	-1.563	0.016	0.308	0.608	1.551	3.579	12.471

### Phylogenetic relationships and divergence time

**Fig 4:**
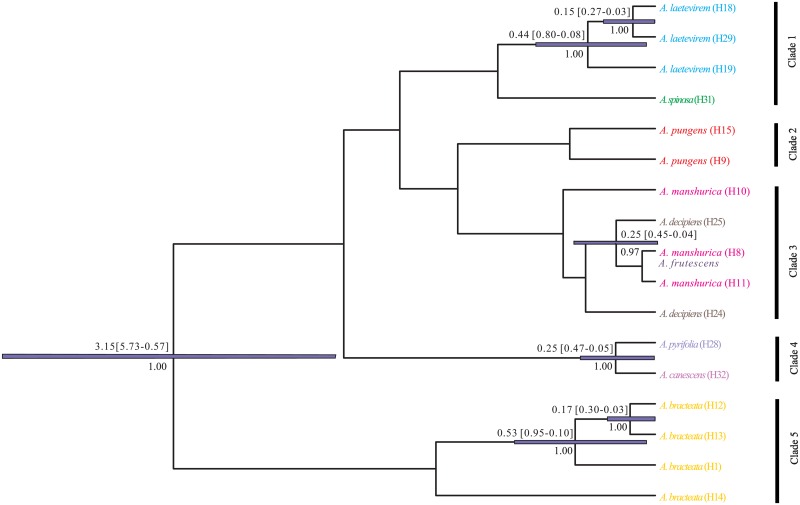
Bayesian phylogenetic tree for *Atraphaxis* based on combined cpDNA matrix. Numbers above branches indicate range of divergence time in millions of years (Ma), and posterior probabilities larger than 0.95 are shown. Clades 1–5 denote five main clades based on identified haplotypes. Haplotype numbers refer to those in Table 1. Colors of each species are consistent with Fig 2.

Based on the Bayesian log-normal relaxed clock method, we obtained an evolutionary tree and divergence time for all of the haplotypes (S2 Fig). However, this tree did not resolve each of the 11 species as reciprocally monophyletic. Some species are sympatric in particular regions (e.g., *A*. *frutescens* and *A*. *laetevirem* are both distributed in Xiaer Lake and in Aretuobie country), and this could induce sympatric introgression among neighboring individuals of different species. For this reason, the phylogenetic relationships of the haplotypes of *Atraphaxis* were well resolved, with the exception of a limited number of haplotypes from these sympatric individuals and two widespread species, *A*. *frutescens* and *A*. *compacta* (S3 Table). Based on the phylogenetic tree, five main haplotype clades can be recognized (Fig 4). Clade 5 comprised all haplotypes of *A*. *bracteata*, a species restricted to the Loess Plateau and separated from other *Atraphaxis* species. Clade 1 contained the haplotypes of *A*. *laetevirem* and *A*. *spinosa*. Clade 3 included haplotypes of two species, *A*. *decipiens* and *A*. *manshurica*. These two species occur in the north of Junggar Basin and the Horqin sandlands. Whereas, haplotype H8 was shared in the species of *A*. *frutescens* and *A*. *manshurica*, suggesting that *A*. *manshurica* has a close relationship with *A*. *frutescens* and may have originated north of Junggar Basin. Clade 4 included the haplotypes of *A*. *canescens* and *A*. *pyrifolia*, which are primarily distributed in the Junggar Basin. Clade 2 contained the haplotypes of *A*. *pungens*.

In the BEAST analysis, the ESS values were over 200 for all nodes. Divergence time among the different haplotypes was estimated between 5.73–0.03 million years ago (Ma). Detailed information concerning relationships among each of the haplotypes separated is depicted in Fig 4.

### Ancestral area reconstructions

**Fig 5:**
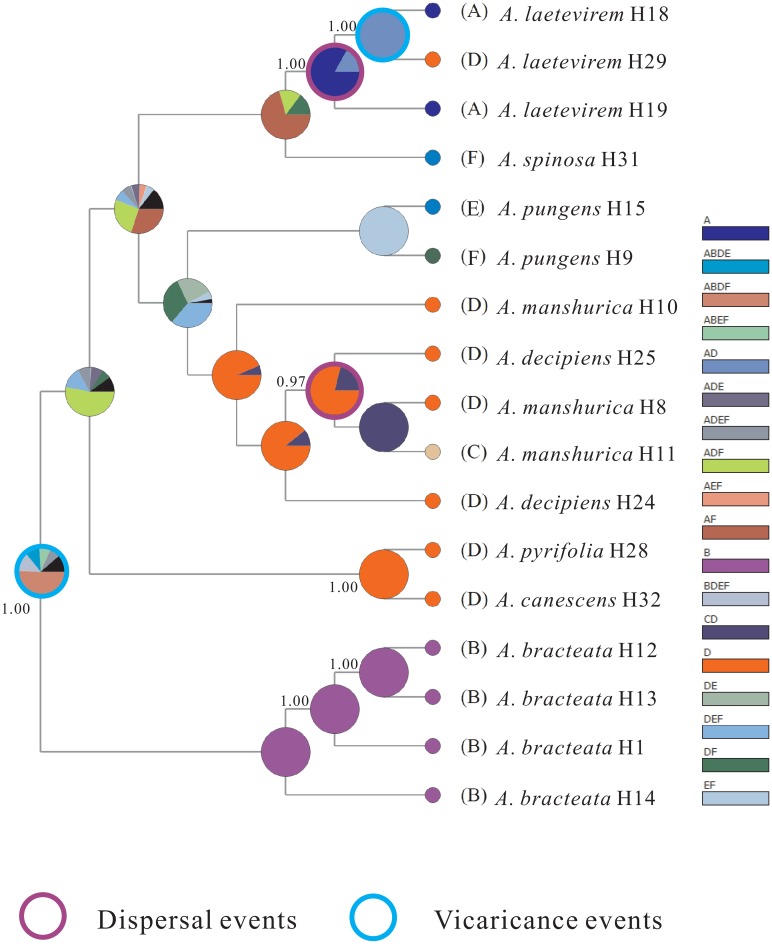
Results of statistical dispersal-vicariance analysis (S-DIVA). Coloured circles correspond to hypothetical ancestral area(s). Letters before names of terminal taxa represent current geographic distributions. Distribution regions: (A) Ili river valley; (B) Loess Plateau; (C) Horqin sandlands; (D) Junggar Basin; (E) Tarim Basin; (F) Qaidam Basin (see Fig 1).

Results of the statistical dispersal-vicariance analysis (S-DIVA) suggest a complex phylogeographic history of the species of *Atraphaxis* in China. Pie charts at nodes indicate the relative frequencies of ancestral area optimization across the entire ancestral area reconstruction tree shown in Fig 5. The Ili valley, Junggar basin, Tarim Basin, and Loess plateau (ABDF, 47.2%) are together resolved to be the most probable ancestral area(s) for *Atraphaxis* in China. Collectively, these regions encompass the entire Western region and some areas of the Central region. Additionally, two dispersal events and two vicariance events are hypothesized based on the S-DIVA analyses, and these events are depicted in Fig 5. The most likely putative dispersal event, recovered by S-DIVA, in *Atraphaxis* is from Junggar Basin (D) to the Horqin sandlands (C), and this was followed by species diversification in these two regions.

## Discussion

Past climatic changes in northern China have resulted in modified ecosystems and species distributions [22]. Indeed, these have affected population contraction and recolonization, range contractions and expansions, and the formation of contact zones between species, all of which have shaped current regional phylogeographic patterns [58]. The phylogeographic patterns of *Atraphaxis* are congruent with those previously described for other arid shrubs and small herbs, although previous studies primarily focused on a single species and a particular geographic area in the arid regions of northern China (e.g., northwestern China, Alashan desert, Horqin sandlands) [7,59,60]. In the present study, we provide a phylogeographic analysis of a species-rich genus throughout its entire geographic range in an arid region.

### Genetic diversity of *Atraphaxis*

The use of cpDNA markers in combination with phylogeographic analyses provide detailed insights into the evolution of species during the late Pliocene and Pleistocene climate shifts. The results obtained from the present study demonstrate phylogenetic relationship and highly structured phylogeographic history in populations and species of *Atraphaxis* (Table 1 and Fig 4). The extreme climate changes that developed during the Pleistocene, and have also occurred since [11], likely played important roles in the diversification of the genus. Cooling sharply increased aridity, in the Chinese deserts, since the late Pliocene [24,25]. Subsequently, geographic isolation and genetic drift with local adaptions for populations (e.g., regarding climate tolerance) may have led to the high levels of genetic differentiation in the species of *Atraphaxis* in northern China. This amount of genetic diversity is important for the maintenance of biodiversity within the genus [61].

As many species inhabit similar ecosystems, admixed species (i.e., species with haplotypes also found in other species) are present in some populations, especially in the Junggar Basin. H4 is shared by three species (*A*. *frutescens*, *A*. *jrtyschensis*, and *A*. *pungens*), and H8 is shared by two species (*A*. *frutescens* and *A*. *manshurica*) (Table 1). This phenomenon can be explained as either retained ancestral polymorphism or introgression. Given the young age and overlapping geographic ranges of the species of the genus, it is likely that both retained ancestral polymorphisms and introgression have played roles in the observed admixture of haplotypes among species (Fig 1). The two processes would function in similar manners. The young age would allow for the observed retention of putative ancestral polymorphisms and the lack of reciprocal monophyly among species, while the sympatric geographic ranges would provide opportunities for introgression, which can lead to genetic admixture. Taking both processes together, it is unsurprising that some widespread haplotypes are present in multiple species.

Western populations within *Atraphaxis* show the largest number of haplotypes and harbour a higher genetic and nucleotide diversity than other areas (Table 1, S1 Table), suggesting that the species in the western region may persist for a longer period of time than species in other areas, such as through multiple glacial cycles. Additionally, the western region includes one of the widespread haplotypes (H4). In contrast, our data demonstrate that *A*. *manshurica* in the Horqin sandlands has the lowest genetic diversity of the three areas, and a small number of haplotypes are present at low frequency (Table 1), with H8 being most common. Interestingly, although H8 is quite common in *A*. *manshurica*, the most eastward species in China, this haplotype is also present in multiple populations throughout the western range of the Chinese species in the genus (*A*. *frutescens* and *A*. *decipiens*), but none in the central part of the distribution. Consequently, it seems likely that *A*. *manshurica* arose via dispersal from western species of *Atraphaxis*. This dispersal event may have occurred via migration along the mountain front and lower montane zones of the slope of Altai Mountains range in China and Mongolia and through the Mongolian Plateau.

### Rapid genetic differentiation in relation to Quaternary climatic events

Based on the BEAST analyses, divergence time between haplotypes of *Atraphaxis* in China is estimated to have occurred 5.73–0.03 Ma (late Pliocene and Pleistocene). This diversification is much more recent than the origin of *Atraphaxis* [33], and this short range of time is likely due, in part, to the inclusion of only Chinese species in the present study. Populations over a limited geographic range may more easily introgress than those from across a larger area, and this can lead to homogenization of the populations and a decreased divergence time. However, the time estimates from BEAST are within the late Pliocene and Pleistocene and fit well with a time when climate changes and aridification began to develop and expand in northwestern and northern China. During the Pleistocene, there were intermittent colder periods as well as aridification in connection with glacial periods [62,63]. Since the early Pleistocene, the climate cooled, and there were several episodes of rapid aridification occurring across northwestern China [64]. The rapid drying of the region and the expansion of desert habitats during the Pleistocene may have acted as an effective stimulus to promote allopatric or parapatric divergence, leading to speciation and intraspecific differentiation within *Atraphaxis*. The consistency between the estimated timeframe of speciation and the glacial and arid expansion history through the Quaternary suggest that mixed and complex climate effects forced rapid speciation, local adaptation and intraspecific differentiation of *Atraphaxis*.

### Ancestral area reconstruction and recent dispersal event

The results of S-DIVA suggest that the ancestor of the species of *Atraphaxis* in China primarily inhabited the Western and Central regions (including the Ili valley, Junggar basin, Tarim Basin, and Loess plateau). The identification of the ancestral area for species of *Atraphaxis* included in the present study coincides with previous hypotheses concerning the origin of the genus. Kransnov considered that the Tertiary mesic montane flora of the Tianshan and other mountains was the area of origin for *Atraphaxis* [65]. Molecular evidence by Zhang et al. [33] indicated that the area of origin for the genus consisted of the Junggar Basin and the uplands of the Pamir-Tianshan-Alatau-Altai mountain chains. The results of these studies are congruent with the central region in the current biogeographic analyses.

It is apparent that *Atraphaxis* in China experienced geographical isolation between the six biogeographic areas (A-F), followed by the detected inter-regional dispersal events (Fig 5). We suggest that the expansion of aridification may have caused these vicariance events. In northern China, *Atraphaxis* grows in areas with extreme drought conditions, such as deserts and semidesert grasslands. In these environments, unfavourable conditions during reproduction would restrict pollination and dispersal capacity of species. This would obstruct gene flow among populations, yet retain gene flow within populations and communities, which can include multiple species of *Atraphaxis*.

According to S-DIVA, a unique dispersal event has been identified from the Junggar Basin (D) to the Horqin sandlands (C). This dispersal event may have occurred via migration along the mountain front and lower montane zones of the Altai Mountains in China and Mongolia and through the Mongolian Plateau. The complexity of topology and melted snows from the peaks of the Altai Mountains may have played an important role in facilitating this migration event. Melted snow in the Altai Mountains would have been an integral source of water in these usually dry areas. During the interglacial period, the climate became warmer, and a greater amount of run-off from melting snow and glacial ice would have infused the lowland areas, making habitats moister [66]. These more favourable environments would have provided better conditions for species survival and range expansion. When populations migrated to semi-arid northeastern China, the altered environment would have provided appropriate conditions for rapid species divergence, including the origin of *A*. *manshurica*. New species resulting from dispersal events may suffer from founder effects, and this seems likely in the case of *A*. *manshurica*. This species is composed of a small number of haplotypes that are also only present in the eastern range of the genus in China.

*Atraphaxis frutescens* has the greatest number of haplotypes, eight (Table 1), and these occur throughout its continuous geographic distribution in the front and lower montane zones of the Tianshan and Altai Mountains, Hexi Corridor, and Qaidam Basin (Table 1, Fig 1). Despite the genetic differentiation of some populations of *A*. *frutescens*, such as those in the east Tianshan Mountains (H3) and the Hexi Corridor (H2), one haplotype dominates. This uniformity probably reflects recent colonization events or the purging of deleterious haplotypes over time. Two of the widespread haplotypes (H3 and H2) also dominate two disjunct populations of *A*. *frutescens*. Between the two regions, we identified a contact zone (LY) in the north of the Hexi Corridor in which mixed haplotypes were present in populations (Table 1 and Fig 1). This contact zone possessed haplotypes (H2 and H3) present in individuals from both regions (i.e., the Tianshan Mountains and Hexi Corridor). Because of the mixed haplotypes, populations in the contact zone display high levels of genetic diversity compared with other populations inhabiting adjacent areas. This contact zone (LY) is located in the transition between the temperate and the warm temperate zones as well as at the intersection of arid and extremely arid regions [67]. This particular geographic position and climatic conditions make this region a significant migratory route for species in adjacent areas.

### Origin and evolution of *A*. *bracteata* in the Loess plateau

From the species included in the BEAST analyses, *A*. *bracteata* is recognized as sister to all other studied species in the genus, and the estimated divergence time for this split is 5.73–0.57 Ma. This is close to the range of the formation of the Tengger Desert, as determined by paleomagnetic data, at approximately 1.8 Ma, which is in the background of a glacial cycle and the uplift of the QTP [68]. Thus, we speculate that *Atraphaxis* existed in the Loess plateau prior to the speciation of *A*. *bracteata*. Subsequently, the expansion of the Tengger Desert split the continuous habitat into two isolated groups. Geographic isolation limited gene flow, and climatic changes may have acted as an effective stimulus to promote divergence between the two groups, leading to the origin of *A*. *bracteata*. The only population in the north of the Hexi Corridor (AX see in Table 1 and Fig 1) consists only of a widely distributed haplotype (H12) of *A*. *bracteata*, and this likely reflects a population expansion event during interglacial cycles of the recent Holocene. During this period, the climate warmed and became wetter, and more snow melted from the Qilian Mountains resulting in large amounts of run-off. This made habitats more suitable for the expansion of populations of *A*. *bracteata*. These findings highlight the predominant role of geographic vicariance in shaping the distribution of *A*. *bracteata*.

## Supporting Information

S1 Fig Geographic distribution of 32 haplotypes recovered from northern China. Pie charts reflect frequency of haplotypes in each population. Haplotype colours correspond to those in panel.
(TIF)

S2 Fig Bayesian phylogenetic tree for *Atraphaxis* based on the entire haplotypes. (TIF)

S1 Table Population code, voucher numbers, haplotype number and distribution, the estimated haplotype diversity (Hd), nucleotide diversity (π, mean number of pairwise differences), and geographic regions (A-F) used in S-DIVA of each *Atraphaxis* population. (DOC)

S2 Table Genbank accession numbers of each haplotype. (DOC)

S3 Table Information for discarded haplotypes in Fig 4. (DOC)
