# Supplementary figures and images for: Phylogeographic History of Atraphaxis Plants in Arid Northern China and the Origin of A. bracteata in the Loess Plateau
Source: PLoS One. 2016 Sep 22;11(9):e0163243. doi: 10.1371/journal.pone.0163243 (PMC5033255; doi:10.1371/journal.pone.0163243)

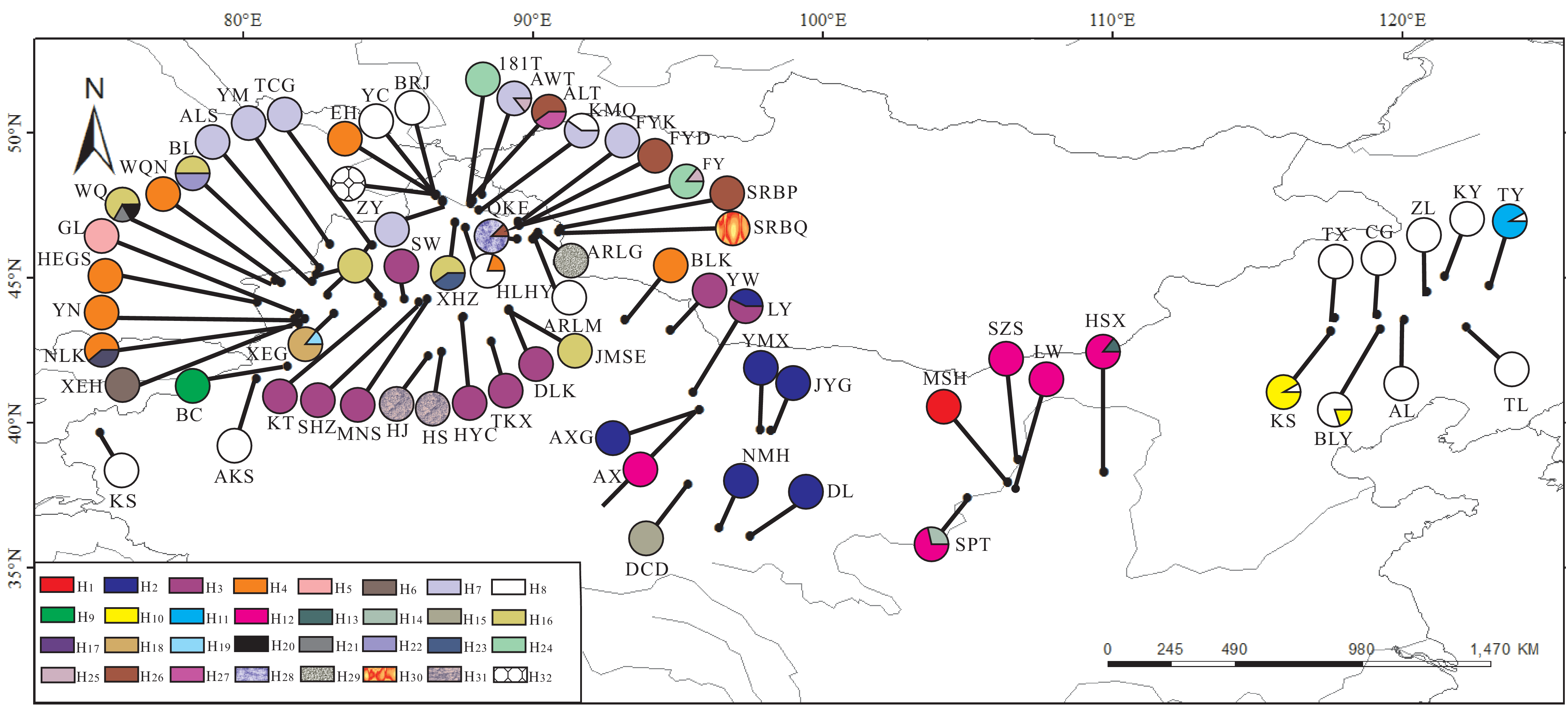

Supplement: S1 Fig — Pie charts reflect frequency of haplotypes in each population. Haplotype colours correspond to those in panel. (TIF) [file pone.0163243.s001.tif]
