# Supplementary material for: Phylogeographic History of Atraphaxis Plants in Arid Northern China and the Origin of A. bracteata in the Loess Plateau
Source: PLoS One. 2016 Sep 22;11(9):e0163243. doi: 10.1371/journal.pone.0163243 (PMC5033255; doi:10.1371/journal.pone.0163243)
Supplement: S1 Table — (DOC) [file pone.0163243.s003.doc]

Table S1 Population code, voucher numbers, haplotype number and distribution, estimated haplotype diversity (Hd), nucleotide diversity (π, mean number of pairwise differences) and geographic regions (A-F) used in S-DIVA of each *Atraphaxis* populations.

| Species | Populations and code | Latitude/longitude | n | haplotype | h(±SD) | π(±SD) (×10^-3^) | mean number of pairwise differences | geographic regions (A-F in S-DIVA) | Voucher numbers |
| --- | --- | --- | --- | --- | --- | --- | --- | --- | --- |
| *A. frutescens* |  |  |  |  | 0.8365±0.0080 | 105.211±55.310 | 6.4178±3.050 |  |  |
|  | 01. JYG | 39.789 / 98.212 | 11 | H2 | 0 | 0 | 0 | B | XJBI:XZJYG1-11 |
|  | 02. YM | 39.815 / 97.842 | 16 | H2 | 0 | 0 | 0 | B | XJBI:XZYM 1-16 |
|  | 03. AXG | 40.500 / 95.738 | 8 | H2 | 0 | 0 | 0 | B | XJBI:XZAXG1-8 |
|  | 04. KT | 44.160 / 84.830 | 9 | H3 | 0 | 0 | 0 | D | XJBI:XZKT1-9 |
|  | 05. SW | 44.322 / 85.584 | 10 | H3 | 0 | 0 | 0 | D | XJBI:XZSW1-10 |
|  | 06. NLK | 43.632 / 82.116 | 18 | H4, H17 | 0.5033±0.0639 | 16.501±13.000 | 1.0065±0.7091 | A | XJBI:XZNLK1-18 |
|  | 07. YN | 43.812 / 81.912 | 6 | H4 | 0 | 0 | 0 | A | XJBI:XZYN1-6 |
|  | 08. GL | 43.625 / 81.793 | 7 | H5 | 0 | 0 | 0 | A | XJBI:XZGL1-7 |
|  | 09. XEH | 43.459 / 81.915 | 11 | H6 | 0 | 0 | 0 | A | XJBI:XZXEH1-11 |
|  | 10. WQN | 44.892 / 81.315 | 18 | H4, H17 | 0.5033±0.0639 | 16.501±13.000 | 1.0065±0.7091 | D | XJBI:XZWQN1-18 |
|  | 11. ALS | 45.369 / 82.626 | 9 | H7 | 0 | 0 | 0 | D | XJBI:XZALS1-9 |
|  | 12. YMX | 46.200 / 83.014 | 12 | H7 | 0 | 0 | 0 | D | XJBI:XZYMX1-12 |
|  | 13. HLHY | 46.770 / 87.666 | 5 | H4, H8 | 0.4000±0.2373 | 6.557±8.338 | 0.4000±0.4351 | D | XJBI:XZHLHY1-5 |
|  | 14. FYK | 46.966 / 89.644 | 8 | H7 | 0 | 0 | 0 | D | XJBI:XZFYK1-5 |
|  | 15. ARLM | 46.499 / 90.097 | 3 | H8 | 0 | 0 | 0 | D | XJBI:XZARLM1-3 |
|  | 16. TKX | 42.861 / 88.659 | 8 | H3 | 0 | 0 | 0 | E | XJBI:XZTKX1-8 |
|  | 17. AKS | 41.573 / 80.463 | 8 | H8 | 0 | 0 | 0 | D | XJBI:XZAKS1-8 |
|  | 18. KS | 39.708 / 75.074 | 11 | H8 | 0 | 0 | 0 | D | XJBI:XZKS1-11 |
|  | 19. SHZ | 44.200 / 86.078 | 5 | H3 | 0 | 0 | 0 | D | XJBI:XZSHZ1-5 |
|  | 20. MNS | 44.308 / 86.335 | 4 | H3 | 0 | 0 | 0 | D | XJBI:XZMNS1-4 |
|  | 21. YC | 47.668 / 86.887 | 3 | H8 | 0 | 0 | 0 | D | XJBI:XZYC1-3 |
|  | 22. HYC | 43.723/ 87.596 | 4 | H3 | 0 | 0 | 0 | D | XJBI:XZHYC1-4 |
|  | 23. DLK | 43.933/ 89.184 | 4 | H3 | 0 | 0 | 0 | D | XJBI:XZDLK1-4 |
|  | 24. NMH | 36.412/ 96.418 | 12 | H2 | 0 | 0 | 0 | F | XJBI:XZNMH1-12 |
|  | 25. DL | 36.121/ 97.511 | 8 | H2 | 0 | 0 | 0 | F | XJBI:XZDL1-8 |
|  | 26. TCGM | 46.168/ 84.454 | 5 | H7 | 0 | 0 | 0 | D | XJBI:XZTCGM1-5 |
|  | 27. BRJ | 47.884/ 86.639 | 5 | H7, H8 | 0.4000±0.2373 | 124.590±81.932 | 7.6000±4.2752 | D | XJBI:XZBRJ1-5 |
|  | 28. LY | 41.102/ 95.542 | 7 | H2, H3 | 0.5714±0.1195 | 28.103±21.244 | 1.7143±1.1313 | B | XJBI:XZLY1-7 |
|  | 29. YW | 43.249/ 94.736 | 7 | H3 | 0 | 0 |  | D | XJBI:XZYW1-7 |
|  | 30. HEGS | 44.198/ 80.483 | 4 | H4 | 0 | 0 |  | A | XJBI:XZHEGS1-4 |
|  | 31. KMQ | 47.781/ 87.869 | 5 | H7, H8 | 0.6000±0.1753 | 186.885±119.742 | 11.4000±6.248 | D | XJBI:XZKMQ1-5 |
| *A. bracteata* |  |  |  |  | 0.3576±0.0911 | 23.992±16.387 | 1.5115±0.9292 |  |  |
|  | 32. MSH | 38.024/ 105.87 | 5 | H1 | 0 | 0 | 0 | B | XJBI:XZMSH1-5 |
|  | 33. HSX | 38.333/ 109.71 | 7 | H12, H13 | 0.2857±0.1964 | 4.608±6.298 | 0.2857±0.3409 | B | XJBI:XZHSX1-5 |
|  | 34. SZS | 38.781/ 106.73 | 7 | H12 | 0 | 0 | 0 | B | XJBI:XZSZS1-7 |
|  | 35. LW | 38.002/ 106.38 | 7 | H12 | 0 | 0 | 0 | B | XJBI:XZLW1-7 |
|  | 36. SPT | 37.460/ 105.00 | 7 | H12, H14 | 0.4762±0.1713 | 61.444±40.237 | 3.8095±2.1778 | B | XJBI:XZSPT1-7 |
|  | 37. AX | 40.500/ 95.738 | 7 | H12 | 0 | 0 | 0 | B | XJBI:XZAX1-7 |
| *A. manshurica* |  |  |  |  | 0.0752±0.0354 | 2.567±3.809 | 0.1720±0.2304 |  |  |
|  | 38. BLY | 43.270/ 119,24 | 10 | H8, H10 | 0.3556±0.1591 | 5.829±6.958 | 0.3556±0.3753 | C | XJBI:XZBLY1-10 |
|  | 39. KQ | 43.200/ 117.55 | 12 | H8, H10 | 0.1667±0.1343 | 2.732±4.432 | 0.1667±0.2401 | C | XJBI:XZKQ1-12 |
|  | 40. TX | 43.680/ 117.68 | 12 | H8 | 0 | 0 | 0 | C | XJBI:XZTX1-12 |
|  | 41. CG | 43.790/ 119.16 | 12 | H8 | 0 | 0 | 0 | C | XJBI:XZCG1-12 |
|  | 42. AL | 43.580/ 120.06 | 12 | H8 | 0 | 0 | 0 | C | XJBI:XZAL1-12 |
|  | 43. ZL | 44.550/ 120.86 | 11 | H8 | 0 | 0 | 0 | C | XJBI:XZZL1-11 |
|  | 44. KY | 45.090/ 121.47 | 12 | H8 | 0 | 0 | 0 | C | XJBI:XZKY1-12 |
|  | 45. TY | 44.790/ 123.01 | 12 | H8, H11 | 0.1667±0.1343 | 14.925±12.140 | 1.0000±0.7224 | C | XJBI:XZTY1-12 |
|  | 46. TL | 43.330/ 122.23 | 11 | H8 | 0 | 0 | 0 | C | XJBI:XZTL1-11 |
| *A. laetevirem* |  |  |  |  | 0.6154±0.0782 | 11.349±10.310 | 0.6923±0.5595 |  |  |
|  | 47. XEG | 43.500/ 81.700 | 7 | H18, H19 | 0.2857±0.1964 | 4.684±6.401 | 0.2857±0.3409 | A | XJBI:XZXEG1-7 |
|  | 48. ARLG | 46.590/ 90.190 | 6 | H29 | 0 | 0 | 0 | D | XJBI:XZARLG1-6 |
| *A. pungens* |  |  |  |  | 0.6277±0.0602 | 29.806±19.897 | 1.8182±1.0878 |  |  |
|  | 49. BLK | 43.600/ 93.170 | 3 | H4 | 0 | 0 | 0 | D | XJBI:XZBLK1-3 |
|  | 50. BC | 42.000/ 81.530 | 8 | H9 | 0 | 0 | 0 | E | XJBI:XZBC1-8 |
|  | 51. DCD | 37.910/ 95.370 | 11 | H15 | 0 | 0 | 0 | F | XJBI:XZDCD1-11 |
| *A. pyrifolia* |  |  |  |  | 0.5267±0.0836 | 80.098±44.464 | 5.4467±2.7132 |  |  |
|  | 52. FYD | 46.860/ 89.540 | 3 | H26 | 0 | 0 | 0 | D | XJBI:XZFYD1-3 |
|  | 53. QKE | 46.390/ 89.470 | 8 | H26, H28 | 0.2500±0.1802 | 40.441±27.233 | 2.7500±1.6257 | D | XJBI:XZQKE1-8 |
|  | 54. SRBP | 46.730/ 90.970 | 9 | H26 | 0 | 0 | 0 | D | XJBI:XZSRBP1-9 |
|  | 55. ALT | 47.380/ 88.150 | 5 | H26, H27 | 0.6000±0.1753 | 59.016±41.962 | 3.6000±2.1896 | D | XJBI:XZALT1-5 |
| *A. jrtyschensis* |  |  |  |  | 0 | 0 | 0 |  |  |
|  | 56. EH | 47.700/ 86.880 | 5 | H4 | 0 | 0 | 0 | D | XJBI:XZEH1-5 |
| *A. canescens* |  |  |  |  | 0 | 0 | 0 |  |  |
|  | 57. ZY | 47.930/ 86.670 | 2 | H32 | 0 | 0 | 0 |  | XJBI:XZZY1-2 |
| *A. decipiens* |  |  |  |  | 0.5417±0.0985 | 54.645±33.148 | 3.3333±1.8052 |  |  |
|  | 58. AWT | 47.920/ 88.230 | 5 | H7, H25 | 0.4000±0.2373 | 131.148±85.916 | 8.0000±4.4830 | D | XJBI:XZAWT1-5 |
|  | 59. 181T | 47.690/ 87.936 | 4 | H24 | 0 | 0 | 0 | D | XJBI:XZ181T1-4 |
|  | 60. FY | 46.960/ 89.500 | 7 | H24, H25 | 0.2857±0.1964 | 9.368±9.780 | 0.5714±0.5208 | D | XJBI:XZFY1-7 |
| *A. compacta* |  |  |  |  | 0.4103±0.0709 | 39.213±23.797 | 2.4704±1.3522 |  |  |
|  | 61. JHN | 44.470/ 82.920 | 16 | H16 | 0 | 0 | 0 | D | XJBI:XZJHN1-16 |
|  | 62. WQ | 44.960/ 81.100 | 6 | H16, H20, H21 | 0.6000±0.2152 | 37.037±27.048 | 2.3333±1.4757 | D | XJBI:XZWQ1-6 |
|  | 63. BL | 44.910/ 82.400 | 6 | H16, H22 | 0.6000±0.1291 | 9.524±10.039 | 0.6000±0.5477 | D | XJBI:XZBL1-6 |
|  | 64. ALSK | 45.160/ 82.530 | 8 | H16 | 0 | 0 | 0 | D | XJBI:XZALSK1-8 |
|  | 65. TCG | 46.170/ 84.450 | 8 | H16 | 0 | 0 | 0 | D | XJBI:XZTCG1-8 |
|  | 66. XHZ | 46.950/ 87.330 | 5 | H16, H23 | 0.6000±0.1753 | 9.524±10.433 | 0.6000±0.5622 | D | XJBI:XZXHZ1-5 |
|  | 67. JMSR | 43.970/ 89.190 | 4 | H16 | 0 | 0 | 0 | D | XJBI:XZJMSR1-4 |
|  | 68. WSC | 44.420/ 84.670 | 4 | H16 | 0 | 0 | 0 | D | XJBI:XZWSC1-4 |
|  | 69. SRBQ | 46.620/ 90.870 | 9 | H30 | 0 | 0 | 0 | D | XJBI:XZSRBQ1-9 |
| *A. spinosa* |  |  |  |  | 0 | 0 | 0 |  |  |
|  | 70. HJ | 42.330/ 86.370 | 8 | H31 | 0 | 0 |  | E | XJBI:XZHJ1-8 |
|  | 71. HS | 42.500/ 86.860 | 7 | H31 | 0 | 0 |  | E | XJBI:XZHS1-7 |
| Total |  |  | 564 |  | 0.8969±0.0069 | 7.234±3.748 | 7.3712±3.4531 |  |  |
| West region |  |  | 359 |  | 0.9138±0.0055 | 7.054±3.666 | 7.1883±3.3775 |  |  |
| Central region |  |  | 103 |  | 0.5877±0.0368 | 127.597±65.858 | 8.5490±3.9840 |  |  |
| East region |  |  | 102 |  | 0.0390±0.0267 | 2.049±3.368 | 0.1373±0.2038 |  |  |
