# Supplementary material for: Phylogeographic History of Atraphaxis Plants in Arid Northern China and the Origin of A. bracteata in the Loess Plateau
Source: PLoS One. 2016 Sep 22;11(9):e0163243. doi: 10.1371/journal.pone.0163243 (PMC5033255; doi:10.1371/journal.pone.0163243)
Supplement: S2 Table — (DOC) [file pone.0163243.s004.doc]

Table S2 The Genbank accession numbers of each haplotype.

| Haplotype | Species | **GenBank acc. numbers** | |
| --- | --- | --- | --- |
|  |  | *psb*K-*psb*I | *psb*B-*psb*H |
| H1 | *A. bracteata* | KX236034 | KX236017 |
| H2 | *A. frutescens* | KM452785 | KM452793 |
| H3 | *A. frutescens* | KM452786 | KM452793 |
| H4 | *A. frutescens*,  *A. pungens*,  *A. jrtyschensis* | KM452787 | KM452793 |
| H5 | *A. frutescens* | KM452789 | KM452793. |
| H6 | *A. frutescens* | KM452790 | KM452793 |
| H7 | *A. frutescens,*  *A. decipiens* | KM452791 | KM452795. |
| H8 | *A. frutescens*,  *A. manshurica* | KR183867 | KR183863 |
| H9 | *A. pungens* | KX236035 | KX236018 |
| H10 | *A. manshurica* | KR183868 | KR183864 |
| H11 | *A. manshurica* | KR183870 | KR183866 |
| H12 | *A. bracteata* | KX236036 | KX236017 |
| H13 | *A. bracteata* | KX236036 | KX236019 |
| H14 | *A. bracteata* | KX236037 | KX236020 |
| H15 | *A. pungens* | KX236038 | KX236021 |
| H16 | *A. compacta* | KX236035 | KX236022 |
| H17 | *A. frutescens* | KM452788 | KM452793 |
| H18 | *A. laetevirem* | KX236039 | KX236023 |
| H19 | *A. laetevirem* | KX236039 | KX236024 |
| H20 | *A. compacta* | KX236035 | KX236025 |
| H21 | *A. compacta* | KX236040 | KX236022 |
| H22 | *A. compacta* | KX236041 | KX236022 |
| H23 | *A. compacta* | KX236042 | KX236022 |
| H24 | *A. decipiens* | KX236043 | KX236026 |
| H25 | *A. decipiens* | KX236043 | KX236027 |
| H26 | *A. pyrifolia* | KX236035 | KX236028 |
| H27 | *A. pyrifolia* | KX236039 | KX236029 |
| H28 | *A. pyrifolia* | KX236044 | KX236030 |
| H29 | *A. laetevirem* | KX236039 | KX236031 |
| H30 | *A. compacta* | KX236044 | KX236032 |
| H31 | *A. spinosa* | KX236045 | KX236022 |
| H32 | *A. canescens* | KX236044 | KX236033 |
