# Supplementary material for: Phylogeographic History of Atraphaxis Plants in Arid Northern China and the Origin of A. bracteata in the Loess Plateau
Source: PLoS One. 2016 Sep 22;11(9):e0163243. doi: 10.1371/journal.pone.0163243 (PMC5033255; doi:10.1371/journal.pone.0163243)
Supplement: S3 Table — (DOC) [file pone.0163243.s005.doc]

Table S3 Information for discarded haplotypes in Figure 4.

| Haplotypes | Species | Populations and code |
| --- | --- | --- |
| H2 | *A. frutescens* | 01. JYG; 02. YM; 03. AXG; 24. NMH; 25. DL; 28. LY |
| H3 | *A. frutescens* | 04. KT; 05. SW; 16. TKX; 19. SHZ; 20. MNS; 22. HYC; 23. DLK; 28. LY; 29. YW; |
| H4 | *A. frutescens*,  *A. pungens*,  *A. jrtyschensis* | 06. NLK; 07. YN; 10. WQN; 13. HLHY; 30. HEGS; 49. BLK; 56. EH; |
| H5 | *A. frutescens* | 08. GL |
| H6 | *A. frutescens* | 09. XEH |
| H7 | *A. frutescens* | 11. ALS; 12. YMX; 14. FYK; 26. TCGM; 27. BRJ; 31. KMQ |
| H16 | *A. compacta* | 61. JHN; 62. WQ; 63. BL; 64. ALSK; 65. TCG; 66. XHZ; 67. JMSR; 68. WSC |
| H17 | *A. frutescens* | 06. NLK; 10. WQN; |
| H20 | *A. compacta* | 62. WQ |
| H21 | *A. compacta* | 62. WQ |
| H22 | *A. compacta* | 63. BL |
| H23 | *A. compacta* | 66. XHZ |
| H26 | *A. pyrifolia* | 52. FYD; 53. QKE; 54. SRBP; 55. ALT |
| H27 | *A. pyrifolia* | 55. ALT |
| H30 | *A. compacta* | 69. SRBQ |
